# The Role of Punicalagin and Its Metabolites in Atherosclerosis and Risk Factors Associated with the Disease

**DOI:** 10.3390/ijms24108476

**Published:** 2023-05-09

**Authors:** Sulaiman Alalawi, Faizah Albalawi, Dipak P. Ramji

**Affiliations:** Cardiff School of Biosciences, Cardiff University, Sir Martin Evans Building, Museum Avenue, Cardiff CF10 3AX, UK; alalawis@cardiff.ac.uk (S.A.); albalawife@cardiff.ac.uk (F.A.)

**Keywords:** atherosclerosis, inflammation, nutraceuticals, oxidative stress, punicalagin, urolithins

## Abstract

Atherosclerotic cardiovascular disease (ACVD) is the leading cause of death worldwide. Although current therapies, such as statins, have led to a marked reduction in morbidity and mortality from ACVD, they are associated with considerable residual risk for the disease together with various adverse side effects. Natural compounds are generally well-tolerated; a major recent goal has been to harness their full potential in the prevention and treatment of ACVD, either alone or together with existing pharmacotherapies. Punicalagin (PC) is the main polyphenol present in pomegranates and pomegranate juice and demonstrates many beneficial actions, including anti-inflammatory, antioxidant, and anti-atherogenic properties. The objective of this review is to inform on our current understanding of the pathogenesis of ACVD and the potential mechanisms underlying the beneficial actions of PC and its metabolites in the disease, including the attenuation of dyslipidemia, oxidative stress, endothelial cell dysfunction, foam cell formation, and inflammation mediated by cytokines and immune cells together with the regulation of proliferation and migration of vascular smooth muscle cells. Some of the anti-inflammatory and antioxidant properties of PC and its metabolites are due to their strong radical-scavenging activities. PC and its metabolites also inhibit the risk factors of atherosclerosis, including hyperlipidemia, diabetes mellitus, inflammation, hypertension, obesity, and non-alcoholic fatty liver disease. Despite the promising findings that have emerged from numerous in vitro, in vivo, and clinical studies, deeper mechanistic insights and large clinical trials are required to harness the full potential of PC and its metabolites in the prevention and treatment of ACVD.

## 1. Introduction

Cardiovascular disease (CVD) remains a leading cause of global morbidity and mortality, accounting for an estimated 17.9-million (31%) deaths according to the World Health Organization [1]. The burden from CVD continues to grow globally due to an increase in risk factors such as obesity, diabetes, high blood pressure, and the western lifestyle [1]. CVD was the cause of most deaths in the UK since the 1950s, but it was overtaken by cancer in 2011 [2]. In 2014, around 27.1% of deaths in the UK were due to CVD [3]. Atherosclerosis is a chronic immune and inflammatory disorder of medium and large arteries involving multiple cell types, including endothelial cells (EC), monocytes, macrophages, smooth muscle cells (SMC), T-lymphocytes, and mast cells, and it is the underlying cause of CVD [4,5,6]. The initiation of atherosclerosis typically starts with the accumulation of low-density lipoproteins (LDL) in the intima of arteries together with other risk factors, which promotes the recruitment of immune cells, including monocytes and lymphocytes. This subsequently leads to the formation of lipid-laden foam cells derived from macrophages and vascular smooth muscle cells (VSMCs) [1]. The retention of an excess amount of intracellular cholesterol in foam cells results in cellular dysfunction and subsequent stress responses, resulting in foam cell death and necrotic core formation [1]. Migrated VSMCs from the media to the intima secrete extracellular matrix (ECM) proteins that form the fibrous cap that covers the necrotic core, and thereby helps in plaque stabilization [1]. Atherosclerosis is a slow and complex disorder that develops over decades and progresses faster with age. Usually, atherosclerosis is asymptomatic until one of the arteries is blocked by thrombosis due to plaque rupture. Therefore, an early diagnosis of atherosclerosis can help via preventive care, including lifestyle modifications, exercise, and changes in diet and medical treatments, to reduce the clinical manifestations of atherosclerotic cardiovascular disease (ACVD), including myocardial infarction (MI), stroke, and ischemic heart diseases [7]. Even though pharmacotherapies, such as statins, have greatly lowered morbidity and mortality rates from ACVD, the persistent risk of primary and secondary major cardiovascular events that occurs after medication, as well as problems such as adverse side effects, has prompted research into alternate prevention and/or treatment approaches [1]. Much of the previous and ongoing research into potential preventative/therapeutic pathways for atherosclerosis have been motivated by the high prevalence of ACVD and the tremendous expense it imposes on healthcare systems [8,9].

## 2. Atherosclerosis

Previously, atherosclerosis was considered as a lipid storage disease. However, extensive research in the last three decades has revealed the significance of low-grade chronic inflammation, thereby explaining the molecular and cellular processes that contribute to atherogenesis [5,6]. Atherosclerosis starts when the various risk factors trigger EC dysfunction/activation that then causes these cells to secrete monocyte chemotactic protein-1 (MCP-1) and other chemokines, which attracts monocytes and other immune cells to the activated endothelium, followed by their adhesion to the endothelial cell surface [10]. Monocytes migrate to the site of inflammation via interactions between receptors on their cell surface and the adhesion molecules expressed on ECs, such as intercellular adhesion molecule-1 (ICAM-1), vascular cell adhesion molecule-1 (VCAM-1), E-selectin and, P-selectin [11], ultimately leading to the accumulation of monocytes in the subendothelial space and their subsequent differentiation into macrophages [8,11,12]. These then uptake oxidized LDL (oxLDL) and other modified LDL, which form in the subendothelial space, via endocytosis mediated by scavenger receptors on their cell surface, including lectin-like oxidized low-density lipoprotein receptor-1 (LOX-1), cluster of differentiation 36 (CD36), and scavenger receptor type 1 (SR-A1), along with other processes such as macropinocytosis and phagocytosis [12]. The modified LDL particles eventually move to the lysosomes as part of the endocytosis process to undergo enzymatic digestion [12]. The released free cholesterol is subsequently esterified to cholesteryl esters (CE) by acyl-CoA acyl transferase-1(ACAT1) and then transferred to the endoplasmic reticulum for storage as lipid droplets [12]. Macrophages with excess CE are called foam cells because of their “foamy” appearance [1]. The accumulation of cholesterol is toxic to the cells and initiates stress responses that ultimately causes them to undergo apoptosis and necrosis leading to the formation of a lipid-rich necrotic core [13]. The lipids in the necrotic core, such as cholesterol crystals, activate the inflammasome pathway leading to the secretion of interleukin (IL)-1β and IL-18, which, together with the production of other inflammatory mediators from various cells present in atherosclerotic plaque, cause a state of low-grade chronic inflammation [8,14,15]. In addition to the innate immune response, cells of the adaptive immune response (e.g., different subtypes of T-cells, B-cells, etc.) play key roles in regulating the chronic inflammation in atherosclerosis [4,5,6,16,17,18]. To counteract the detrimental changes and to maintain homeostasis, necrotic cells are cleared in the early stages of the disease by phagocytosis in a process called efferocytosis [19,20]. However, in advanced lesions, efferocytosis becomes ineffective as the plaques progress. Hence, there is an increase in the accumulation of apoptotic cells [1,19,20]. SMCs undergo a phenotypic shift (quiescent state to proliferative state) and migrate from the tunica media to the intima [1]. Some of migrated SMCs also become foam cells by the uptake of modified LDL. They also secrete ECM proteins, which contribute to the formation of a fibrous cap over the necrotic core [1]. As the formation of the fibrous cap progresses, a stable atherosclerotic lesion is formed [1]. Subsequently, ECM proteins are degraded by protease enzymes, particularly matrix metalloproteinases produced by macrophages, foam cells, and other cells during chronic inflammation [1]. This initiates the destabilization of the plaque, which ultimately leads to its rupture, thrombosis, and ACVD, including MI and cerebrovascular accidents [1,21]. Some of the risk factors for, and key cellular processes in, atherosclerosis are addressed below in more detail.

### 2.1. Risk Factors for Atherosclerosis

There are many risk factors for ACVD that are generally classified as modifiable and non-modifiable [22]. The former includes dyslipidemia, smoking, hypertension, diabetes, and obesity, whereas the latter includes age, male gender, and genetic predispositions such as familial hypercholesterolemia and Tangier disease [23]. Obesity, diabetes, and hypertension are all known to contribute to ACVD and increase its burden, but recent studies have also revealed an important role of non-alcoholic fatty liver disease (NAFLD) [24,25,26]. This is associated with an accumulation of lipids in the liver, which is caused by an imbalance between lipid uptake, storage, and utilization [27] in individuals who drink little or no alcohol [28]. Thus, NAFLD is characterized by an increase in the uptake of free fatty acids (FFA) by the liver and a decrease in their utilization for energy production, which initially leads to lipid accumulation that can eventually progress to nonalcoholic steatohepatitis (NASH) if untreated [25]. Atherosclerosis and NAFLD are closely linked through multiple mechanisms and risk factors such as diabetics, obesity, and metabolic syndromes [29]. Additionally, NAFLD is associated with increased oxidative stress and inflammation, which is also known to contribute to the development of atherosclerosis [30]. In addition, NAFLD is linked to changes in lipid metabolism that may have a direct role in the onset of atherosclerosis. Thus, triacylglycerol (TG) and LDL, known risk factors for atherosclerosis, are elevated in individuals with NAFLD, which is often also associated with reduced levels of the protective high-density lipoprotein (HDL) [31].

### 2.2. Role of Oxidative Stress in Atherosclerosis

An imbalance between the production of reactive nitrogen species (RNS) and reactive oxygen species (ROS), together with an absent or poor antioxidant system, leads to oxidative stress [32]. ROS can be of exogenous or endogenous origin, while the mitochondrial respiratory chain is the main endogenous source of ROS [33]. During the early stages of atherosclerotic lesion formation, enzymes such as nitric oxide synthase and xanthine oxidase contribute to oxidative stress [34], which is also associated with local inflammation, endothelial dysfunction, SMC proliferation, and plaque formation [35]. ROS also leads to the production of growth factors and mitogens by various cell types that then contributes to the stimulation of cell proliferation in early atherosclerotic lesions [36]. Oxidative stress also contributes to DNA instability, DNA mutations, dysfunction in the products of repair genes, and hyper-methylation [37]. Thus, ROS is considered as a major contributing factor in LDL oxidation and various signaling processes in the pathogenesis of atherosclerosis.

### 2.3. Role of Macrophages in Atherosclerosis

Macrophages are typically produced via differentiation of monocytes by macrophage-colony stimulating factors [38]. They are cells of the innate immune system that play pivotal roles in mounting an immunological reaction against foreign antigens or pathogens, including viruses and bacteria, and contribute to the regulation of inflammatory responses [39]. Several phenotypes of macrophages have been identified, with the two most common and well-documented being M1 (pro-inflammatory) and M2 (anti-inflammatory) [6,40]. M1 macrophages express several pro-inflammatory mediators, including IL-1, IL-6, ROS, RNS, and tumour necrosis factor (TNF)-α, while M2 macrophages are associated with the resolution of inflammation and express IL-10, the mannose receptor CD206, and arginase 1 [41]. Macrophages play a crucial role in atherosclerosis [8,42,43,44], where they are involved in all key processes, including foam cell formation, the development of lipid-rich necrotic core, the orchestration of the inflammatory response, and plaque rupture [12]. Excessive macrophage foam cell formation occurs during atherosclerosis because of an enhanced uptake of modified lipoproteins by scavenger receptors-mediated endocytosis, macropinocytosis and phagocytosis, and defective cholesterol efflux [1,12].

### 2.4. Role of Cytokines in Atherosclerosis

Cytokines are a class of small mediator proteins or glycoproteins that are secreted by many cells, including ECs, VSMCs, monocytes, macrophages, and T cells, in response to inflammation, infection, and other stimuli [6,8,45]. Cytokines are a complex series of proteins that comprise more than 100 released molecules that may be classified into various classes, including transforming growth factors, TNFs, colony-stimulating factors, ILs, interferons (IFNs) and chemokines [8,46]. The balance between pro-inflammatory and anti-inflammatory cytokines is crucial in the maintenance of cardiovascular health and in atherosclerosis, the balance is tipped towards the accumulation of pro-inflammatory cytokines [8,47]. As discussed above, cytokine-induced EC activation plays an important role in endothelial dysfunction and is followed by an increase in the recruitment and migration of immune cells into atherosclerotic sites [48]. The function of VSMCs is also regulated by cytokines that modulate their proliferation, migration, senescence, and phenotypic conversion [49].

### 2.5. Lysosomal Dysfunction in Atherosclerosis

As macrophages are involved in modified LDL uptake in early atherosclerotic lesions, their failure to digest the accumulated lipids contributes to the development and complexity of the disease [50]. Lysosomes play a crucial role in the maintenance of metabolic homeostasis of cells by degradation and sequestration of macromolecules [51], and lysosomal dysfunction is associated with sterile inflammation in atherosclerosis [52,53]. Macrophages develop features of lysosomal dysfunction following an exposure to atherogenic lipids [54]. Lysosomal stress can also activate transcription factor EB (TFEB), which acts as a main regulator of lysosomal biogenesis and function [55]. TFEB is regulated by multiple signaling pathways and can modulate several processes that are important in atherosclerosis, including lipophagy, autophagy, lipolysis, and inflammation [56,57,58,59,60,61]. Thus, lysosomal biogenesis in macrophages stimulated by TFEB may serve as a protective factor for atherosclerosis [62]. THP-1 macrophages treated with modified LDL develop lysosomal dysfunction [63], and mitochondrial ROS-induced lysosomal dysfunction promotes inflammation by contributing to M1 macrophage polarization [64]. Overall, lysosomal dysfunction is one of the multiple causes of atherosclerosis progression [54].

### 2.6. Reverse Cholesterol Transport and Atherosclerosis

Reverse cholesterol transport (RCT) is mediated by HDL, where this lipoprotein transports cholesterol from peripheral tissues back to the liver, where some of it is excreted via the bile system [12]. Hepatocytes and enterocytes are involved in the formation of nascent HDL that matures through the binding of phospholipids and free cholesterol effluxed from foam cells by ATP-binding cassette (ABC) transporters [12,65]. ABCA1 mediates cholesterol efflux to apolipoprotein (Apo) A1 present in HDL, whereas ABCG1 stimulates cellular cholesterol efflux to HDL [66]. Additionally, ABCA1 trafficking between the late endocytic vesicles and the cell surface is required for cholesterol efflux from endosomal/lysosomal compartments to lipid-free ApoA1 [67]. Furthermore, the intracellular sterol transporter ABCG1 stimulates cholesterol trafficking from the ER to the plasma membrane [68]. In addition to ABCA1 and ABCG1, the passive diffusion of cholesterol and scavenger receptor class B type 1 (SR-B1) enables the binding of lipids to HDL [69]. After the binding of cholesterol to HDL, the lecithin cholesteryl acyltransferase enzyme initiates esterification of the acquired cholesterol to form CE, forming mature HDL [70]. Hepatic lipase and endothelial lipase, respectively, mediate the remodeling of HDL particles via the hydrolysis of phospholipids and TG present in the lipoprotein [71]. The CE in the HDL core is transferred by cholesteryl ester transfer protein (CETP) to other lipoproteins, such as LDL, for removal by the liver through the low-density lipoprotein receptor (LDLr) and, ultimately, in the case of HDL, via SR-B1 [72]. CE is hydrolyzed in the liver, and free cholesterol is converted to bile acids that are used for the emulsification of lipids during intestinal digestion. The majority of bile is reabsorbed but some is lost from the body via the faeces [23,73].

## 3. Current Pharmacotherapies for Atherosclerosis

Statins, competitive inhibitors of 3-hydroxy-3-methylglutaryl coenzyme A reductase, which catalyzes a rate-limiting step in the biosynthesis of cholesterol in the mevalonate pathway, are gold standard therapies against ACVD through reductions in plasma cholesterol levels [1,9,23]. The inhibition of the mevalonate pathway also decreases the levels of isoprenoid metabolites, geranylgeranyl pyrophosphate, and farnesyl pyrophosphate, that critically modify small-signaling G proteins that are involved in the regulation of numerous cellular functions, including survival, proliferation, and migration. Hence, these contribute to the pleiotropic actions of statins, such as anti-inflammatory actions and the attenuation of EC dysfunction [74,75]. However, statins are associated with various adverse side effects, such as myalgias, hepatic abnormalities, rhabdomyolysis, and diabetes in some cases [1,9,76]. Some emerging recent therapies target the intestinal absorption of dietary cholesterol (e.g., ezetimibe), plasma cholesterol levels (e.g., monoclonal antibodies against proprotein convertase subtilisin/kexin type-9, which prevent the degradation of LDLr, and bempedoic acid, which inhibits another enzyme in the cholesterol biosynthetic pathway), or inflammation (e.g., monoclonal antibody against the pro-inflammatory cytokine IL-1β and colchicine that inhibits the inflammasome pathway involved in the production of pro-inflammatory cytokines) [1,9]. Again, these agents have various issues, such as side effects (e.g., targeting inflammation makes individuals more prone to infections), costs associated with monoclonal antibodies as therapies, residual risk for disease, and non-compliance [1,9]. Many other promising agents have been unsuccessful in clinical targets because of side effects and off-target effects, including inhibitors of CETP, nicotinic acid, and its derivatives, such as niacin, and methotrexate [1,9,77]. Therefore, it is essential to find alternative preventive and therapeutic agents with better safety profile and less adverse side effects in the treatment ACVD.

## 4. Potential Nutraceutical Therapies for Atherosclerosis

One potential avenue being investigated for the prevention and treatment of ACVD is nutraceuticals or natural products with health benefits beyond their nutritional value, particularly those from phenolic-rich diets that are abundant in anti-oxidant and anti-inflammatory components [78,79,80,81]. The increased consumption of polyphenolic-rich fruits and vegetables has been linked to health benefits related to cardiovascular function. For example, it has been found that ACVD risk factors are reduced by 46% in individuals consuming polyphenol-rich diets [82]. Other studies have revealed that polyphenols can inhibit platelet aggregation [83], improve plasma lipid profile and inflammation markers [84], and help in maintaining endothelial function [85]. Pomegranate (*Punica granatum*) is rich in many polyphenol compounds, including anthocyanins and anthoxanthins, such as catechins, punicalagin (PC), ellagic acids (EA), gallic- and ellagi-tannins [86]. Both hydrolyzable ellagitannins (ET) and EA are potent antioxidants and are involved in the protection against atherogenesis [87]. PC is the main polyphenol ET in pomegranate and is responsible for its high antioxidant and anti-atherogenic activities [88]. Pomegranates cultivated in the Mediterranean, Middle East, India, China, Japan, and the United States have been reported as one of the fruits that contains the highest antioxidant, anti-atherogenic, anti-cancer, and anti-inflammatory components [89]. However, these potent effects are attributed to the highest concentration of polyphenols in pomegranates, including flavonoids, ET (e.g., PC, EA), and anthocyanins [90].

### 4.1. Bioavailability and Metabolism of Punicalagin and Its Metabolites

**Figure 1:**
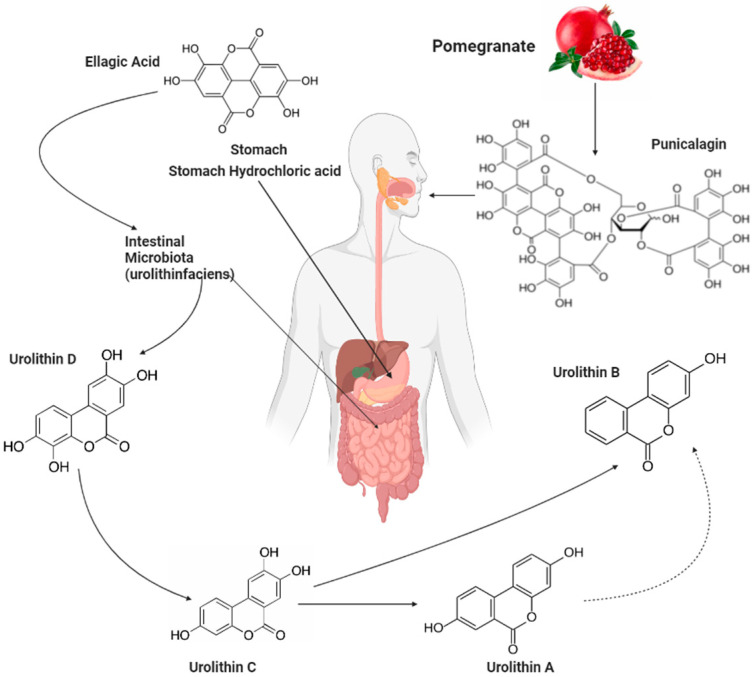
Transformation of punicalagin to urolithins. Food-derived ellagitannins, such as punicalagin in pomegranates, are first hydrolyzed in the stomach to produce ellagic acid. This then undergoes a series of transformation by the gut microbiota to produce the different urolithins with urolithin-A and -B being two key final products. Figure created using Biorender.com, accessed on 28 March 2023.

ET is a family of polyphenols present in nuts and fruits, such as walnuts, strawberries, and pomegranates [91]. Its consumption has been widely studied in relation to health promotion [92,93] due to their beneficial anti-inflammatory, anti-atherogenic, and antioxidant properties, among others [94,95]. The most abundant of these ET polyphenols are PC and EA [96]. However, PC and EA are not readily detected in human tissues or plasma after consumption of a high amount of pomegranate products [97]. It is now known that after the ingestion of pomegranate or pomegranate-based products, the ET polyphenols are poorly absorbed by the intestine because they are large (e.g., molecular weight of 1084.71 g/mol for PC) and hydrophobic, and the gut microbiota transforms them into potent metabolites called urolithins (Uro) [92,98] (Figure 1). Due to the poor bioavailability of PC and EA and extensive gut catabolism, it has been suggested that Uro, rather than EA and PC, are the actual bioactive molecules [99,100,101]. The circulation and distribution of PC, EA, and their metabolites have been studied in different tissues in human and animals, including pigs, sheep, birds, rodents, and insects [102,103]. In addition, Uro have been found to significantly accumulate in plasma and tissues [92,104,105]. As shown in Figure 1, PC from pomegranates and other sources is mainly hydrolyzed into EA in the acidic environment of the stomach. EA then undergoes a series of metabolic transformations by the gut microbiota to form Uro, with UroA and UroB being the two key final products.

### 4.2. Molecular Mechanisms Underlying the Beneficial Actions of Punicalagin and Its Metabolites in Atherosclerosis and Risk Factors Associated with the Disease

**Figure 2:**
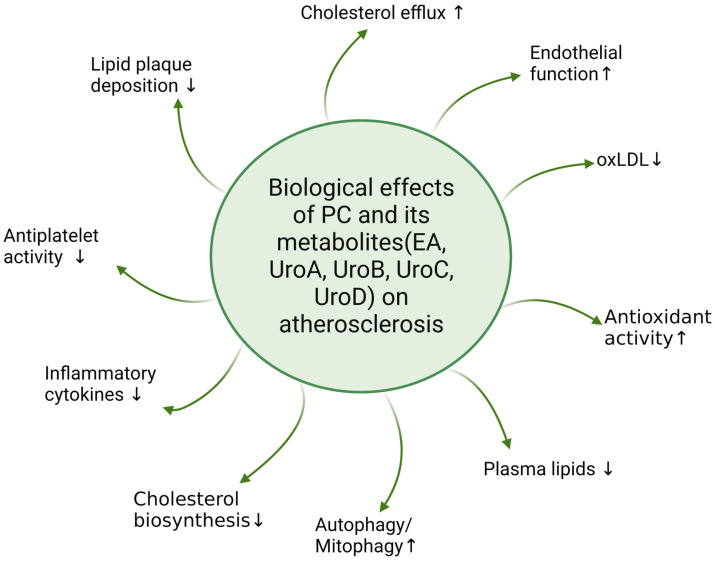
The biological effects of PC and its metabolites on atherosclerosis. ↑, increased/improved; ↓, decreased; EA, ellagic acid; oxLDL, oxidized low density lipoprotein; PC, punicalagin; UroA, urolithin A; UroB, urolithin B; UroC, urolithin C; UroD, urolithin D. Figure created using Biorender.com, accessed on 28 March 2023.

Some of the key anti-atherogenic actions of PC and its metabolites are indicated in Table 1 and summarized in Figure 2. These include antioxidant activities, effects on lipoprotein oxidation and metabolism, lipid accumulation and foam cell formation, and cytokine expression and inflammation together with impacts on disease-associated risk factors and the gut microbiota. These are addressed below in more detail.

#### 4.2.1. Punicalagin and Metabolites as Antioxidants

Polyphenol compounds exert their antioxidant activity primarily via radical scavenging that, in vitro, involves the donation of an H atom or electron from their hydroxyl group to the free radical [117]. PC is known to have the most potent antioxidant activities when compared to other polyphenols [118]. The supplementation of pomegranate juice and pomegranate fruit extract rich in PC can significantly increase endothelial nitric oxide synthase (eNOS) activity, leading to the attenuation of pro-atherogenic-perturbed shear stress induced in vitro in human coronary ECs and in vivo in hypercholesterolemic mice [119]. Endothelial dysfunction due to high glucose is linked to the elevated generation of ROS and the treatment of human aortic ECs, as well as intact rat aortas with high glucose (30 mM) and EA, resulted in a significant decrease in ROS levels and improved the impaired vascular relaxation induced by the high glucose via the downregulation of nicotinamide adenine dinucleotide phosphate (NADPH) oxidase 4 (NOX4) and the inhibition of extracellular signal-regulated kinase (ERK) 1/2-signaling pathways [120]. ROS can also cause cellular damage, particularly to DNA, RNA, proteins, and lipids, which can then lead to inflammation [121]. Uros-reduced ROS generation in both short- and long-term incubations by decreasing catalase, glutathione peroxidase, and superoxide dismutase enzymes in Caco-2 enterocytes, thereby preventing oxidative cellular damage [122]. In addition, the pre-treatment of human umbilical cord endothelial cells (HUVEC) with EA followed by exposure to oxLDL significantly attenuated ROS production, cytotoxicity, and apoptotic features by modulating eNOS and phosphoinositide 3-kinase pathways [123]. UroB showed antioxidant properties by reducing NADPH oxidase subunit expression and intracellular ROS production and inducing the antioxidant hememoxygenase-1 expression by nuclear factor erythroid 2-related factor 2 (Nrf2)/antioxidant response element signaling in BV2 microglial cells [124]. Kelch-like ECH-associated protein 1 (Keap1) represses Nrf2 activity via multiple mechanisms; this can be interrupted by many proteins, including a ubiquitin-binding protein p62 [125]. UroB protected against myocardial ischemia/reperfusion injury via an increased accumulation of p62 and its subsequent interaction with Keap1, thereby resulting in protection against superoxide production and apoptotic cell death [126]. UroA also demonstrated anti-oxidative and neuroprotective actions in Alzheimer’s disease by inhibiting high glucose-induced amyloidogenesis produced by mitochondrial calcium dysregulation and mitochondrial ROS accumulation [127].

#### 4.2.2. Effects of Punicalagin and Its Metabolites on Lipoprotein Metabolism and Lipid Homeostasis

Lipoproteins, including LDL, HDL, and their oxidatively modified forms, play vital roles in cholesterol metabolism and associated disorders [22]. In addition, foam cell formation during atherosclerosis is controlled by the uptake, intracellular metabolism, and efflux of cholesterol in macrophages and VSMC [12]. Furthermore, lipid metabolism in other tissues (e.g., liver, adipose tissue) impacts lipid homeostasis in atherosclerotic plaques [12]. Polyphenols such as PC have many protective actions against lipid and lipoprotein homeostasis, as well as processes regulated by their dysfunction [128,129]. Thus, a randomized, double-blinded, placebo-controlled, crossover trial performed on 67 healthy adults for 20 weeks to evaluate the effects of hydroxytryrosol and PC on early atherosclerosis-associated markers showed that supplementation with these two polyphenols exerted anti-atherosclerotic effects by improving blood pressure, endothelial function, and decreasing the levels of circulating oxLDL [88]. In an in vivo study, Wistar rats were fed with a high-cholesterol diet supplemented with Vitamin D3 and subjected to the balloon injury of the aorta. UroA (3 mg/kg/day) produced a significant improvement in the plasma lipid profile and Angiotensin II levels together with aortic lesions compared with the control group [111]. UroB also decreased lipid plaque deposition in Apolipoprotein E-deficient (ApoE^−/−^) mice and enhanced macrophage cholesterol efflux through the induced expression of SR-BI and ABCA1 [107]. The combination of pomegranate and dates in ApoE^−/−^ mice reduced plasma cholesterol and TG levels associated with increased paraoxonase activity and lipid peroxide content in the aorta and produced significant decrease in oxidative stress, cholesterol content, and LDL uptake in peritoneal macrophages [130]. In in vitro studies, the treatment of murine J774A.1 macrophages with PC-enhanced statin-mediated cholesterol biosynthesis and protected against foam cell formation [110,130]. EA also decreased the oxLDL-mediated foam cell formation in J774A.1 macrophages and enhanced cholesterol efflux from foam cells [109].

The impact of PC, EA, and its metabolites on lipid metabolism is not just restricted to macrophages but extends to other cellular systems. Thus, UroA, UroB, and UroC reduced TG accumulation and fatty acid oxidation in adipocytes and hepatocytes [131]. EA and UroA attenuated lipid accumulation in 3T3–L1 adipocytes through the regulation of glucose Transporter Type 4 and adiponectin [132]. In streptozotocin-induced Type 1 diabetes mellitus in rats, EA prevented hepatic lipid accumulation and reduced both hepatic and plasma levels of TG, cholesterol, and FFAs by activating AMP-activated protein kinase [133]. Furthermore, EA decreased plasma cholesterol and TG levels and increased fecal bile acid excretion in hamsters. This was associated with an increased expression of liver X receptor-α, peroxisome proliferator-activated receptor -γ, and -α, together with their downstream target gene ABCA1 [134].

#### 4.2.3. Effects of PC and Its Metabolites on Inflammation and Expression of Cytokines

Anti-inflammatory effects of PC and its metabolites are widely highlighted in the literature [135,136,137]. Thus, UroA significantly attenuated the production of pro-inflammatory mediators in lipopolysaccharide (LPS)-stimulated RAW264 macrophages, leading to the inhibition of Akt and c-Jun N-terminal kinase phosphorylation and nuclear factor kappa-light-chain-enhancer of activated B cells (NF-κB) and activator protein-1 activation [138]. UroA also inhibited TNF-α-induced expression of both MCP-1 and IL-8 in human aortic ECs together with their migration and adhesion to monocytes [94]. In addition, an in vitro study in THP-1 macrophages, in which IFN-γ was used to induce the expression of several pro-inflammatory genes, in particular ICAM-1 and MCP-1, PC significantly inhibited their expression together with MCP-1-induced monocytic migration [139]. UroA also reduced the expression of various inflammatory factors in response to the oxLDL stimulation of human artery ECs, such as MCP-1, ICAM-1, TNF-α, and IL-6. Consequently, it attenuated the adhesion of monocytes to these cells [94,116]. In another study, in human placenta, visceral adipose tissue and subcutaneous adipose tissue explants, treatment with PC and curcumin significantly suppressed the TNF-α-induced expression of chemokines [C-C motif ligand (CCL)2-5, C-X-C motif ligand (CXCL)1, CXCL5, CXCL8] and pro-inflammatory cytokines (IL-1α, IL-1β, IL-6) [140]. EA also inhibited oxLDL- and IL-1 β-mediated activation of NF-κB, as well as downstream targets, such as cytokines and adhesion proteins, in HUVEC [114,141]. UroB also inhibited the production of pro-inflammatory cytokines and increased an anti-inflammatory cytokine IL-10 in LPS-stimulated BV2 microglial cells [124].

As detailed above, in vivo, UroB attenuated atherosclerosis, an inflammatory disorder, in ApoE^−/−^ mice [107]. In male Balb/c mice fed a HFD for 12 weeks and given PC subcutaneously in the last four weeks, there was a significant improvement in HDL anti-inflammatory properties and other anti-inflammatory parameters compared to the control [137]. The oral supplementation of EA also downregulated the expression of pro-inflammatory cytokines IL-1β, IL-6, and TNF-α in isoproterenol-treated rats and protected against cardiac damage [142]. The effect of UroB after MI was investigated in vivo using adult male Sprague Dawley rats and revealed cardioprotective effects through the control of inflammation and cardiac fibrosis [115].

#### 4.2.4. Effects of PC and Its Metabolites on Other Pathologies That Impact Atherosclerosis

**Figure 3:**
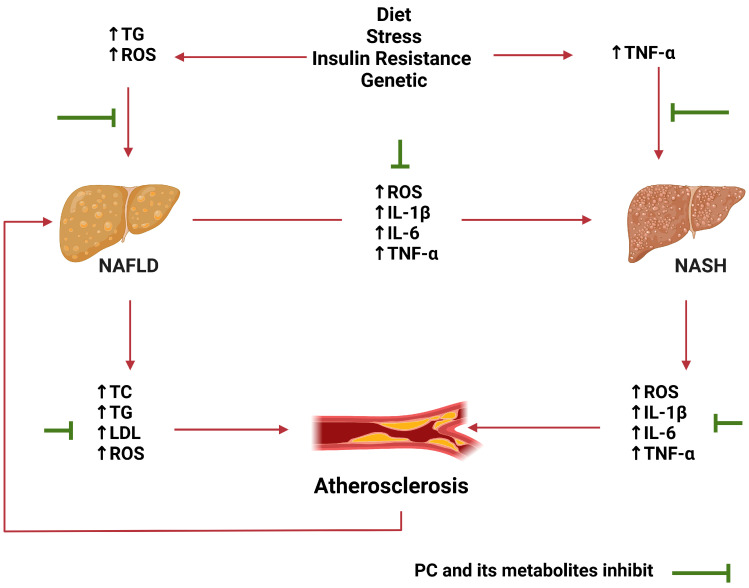
Effect of PC and its metabolites on various factors influencing atherogenesis and non-alcoholic fatty liver disease progression. Both atherosclerosis and NAFLD/NASH are caused, and exacerbated by, a number of similar factors, including diet, stress, insulin resistance and genetic disorders, combined with an increase in triacylglycerol, reactive oxygen species, and tumour necrosis factor-α levels. These can then cause the development of NAFLD/NASH in the liver, which further contributes to the production of pro-inflammatory cytokines, such as interleukin-1β and -6, or elevated triacylglycerol and reactive oxygen species, which can all contribute to the development of atherosclerosis. PC and its metabolites can decrease the impact of these factors and represent the prevention or therapeutic approach to NAFLD/NASH and atherosclerosis. ↑, increased; IL, interleukin; LDL, low-density lipoprotein; NAFLD, non-alcoholic fatty liver disease; NASH, non-alcoholic steatohepatitis; ROS, reactive oxygen species; TG, triacylglycerol; TNF, tumour necrosis factor. Created using BioRender.com, accessed on 28 March 2023.

Other inflammatory disorders, such as rheumatoid arthritis, asthma, Type 2 diabetes, and NAFLD, also impact atherosclerosis [25]. PC and its metabolites also affect these disorders and, therefore, atherosclerosis; this is illustrated effectively in Figure 3 in relation to NAFLD. Thus, in an in vivo study, EA improved hepatic steatosis and the plasma lipid profile in the KK-A(y) mice that were fed HFD as a model for obese Type 2 diabetes [143]. PC (20 mg/kg body weight/day) protected against HFD and streptozotocin-induced diabetic liver injury in C57BL/6 mice via the activation of antioxidant enzymes and upregulation of mitophagy [144]. UroA also decreased ROS levels in HepG2 cells, together with the expression of NF-κB p65 and other inflammatory markers, and improved antioxidant activities [145]. It has been reported that UroA, UroC, and UroD also increase fatty acid oxidation and attenuate TG accumulation in hepatocytes and adipocytes [131]. In relation to other inflammatory disorders, EA was found to inhibit IL-1β-induced activation of NF-κB signaling and several downstream genes in human chondrocytes and protected in a surgical DMM (destabilization of the medical meniscus) model of osteoarthritis [146]. In an ovalalbumin-induced mouse asthma model, EA also inhibited NF-κB activation and the development of airway hyper responsiveness (e.g., lung eosinophilic inflammation and goblet cell hyperplasia) [147].

#### 4.2.5. The Impact of PC and Its Metabolites on the Gut Microbiota

Polyphenols can influence the gut microbiota in a manner that encourages the growth of beneficial bacteria while inhibiting the growth of harmful bacteria [148]. In addition, the microbiome can influence polyphenols to become more bioavailable via metabolism into new metabolites [149]. For example, the gut bacteria *Gordonibacter pamelaeae* and *Gordonibacter urolithinfaciens* have shown the potential to biotransform EA to urolithins [150]. PC can alleviate insulin resistance by regulating gut microbiota and autophagy [151]. Some of the actions of PC and its metabolites may potentially be mediated via the modulation of the gut microbiota. Thus, UroA and UroB possess anti-obesity properties in a HFD-induced rat model of obesity via modulation of the gut microbiota [152]. Polyphenols often cause the production of short chain fatty acids [153,154], such as propionate and butyrate, which then prevents ACVD [155,156].

## 5. Conclusions

PC and its metabolites have protective actions on atherosclerosis and other inflammatory disorders via multiple mechanisms, including antioxidant and anti-inflammatory properties, together with the modulation of the gut microbiota. However, studies on the role of PC and its metabolites in mouse models of atherosclerosis have been limited to PC and UroB [107,130]. In addition, these studies have been rather restricted to monitoring only some parameters (e.g., plaque lipid content). More detailed studies are required that investigate the effects of PC and its metabolites on plaque burden, lipid content, and cellularity (e.g., levels of macrophages, T-cells and various subtypes, other immune cells, smooth muscle cells etc), using a combination of histological and immunohistological analyses, similar to those carried out on other nutraceuticals [157]. Such studies can provide key information on whether PC and its metabolites can dampen plaque inflammation and stabilize existing plaques, thereby informing on mechanisms of actions and therapeutic potential. The studies in such models can be extended to plasma lipid profile, immune cell profile in peripheral blood, bone marrow, spleen, and thymus together with other tissues (e.g., liver in relation to NAFLD). Gene expression analysis using arrays, RNA-sequencing (RNA-seq), and single-cell RNA-seq on the aorta and the liver [157,158,159] can provide insights into pathways regulated by PC and its metabolites in atherosclerosis and NAFLD, and whether there are common genes/pathways regulated by different agents. The identification of key pathways can also form the foundation of drug discovery programs to screen for potent agonists. More mechanistic insights can also be obtained using the full range of in vitro assays on all the different cell types present in atherosclerotic plaques, including macrophages, ECs, and SMCs [160]. Such assays include cell proliferation and migration, foam cell formation, inflammasome activation, endothelial cell dysfunction, and SMC phenotypic shift. The in vitro and in vivo assays should employ the full range of physiological doses and can be extended further to investigate whether PC and its metabolites can also cause a regression of existing atherosclerotic plaques in mouse model systems [159]. Combining agents may provide additional insights on the existence of any synergistic or antagonistic actions [161]. Such studies can then form the foundations for large clinical trials; initial studies in humans have already shown promise in relation to improved mitochondrial and cellular health [112]. Given the existence of co-morbidities, studies on PC and its metabolites should ultimately be extended beyond atherosclerosis and NAFLD to include neurological disorders, metabolic syndrome, diabetes, and obesity.

## Figures and Tables

**Table 1:** The main biological effects of PC and its metabolites in vitro and in vivo.

Markers	Agent	Duration	Model	Reference
oxLDL levels or oxLDL-mediated responses ↓	PC (195 mg/day)	20 weeks	Human: healthy individuals, 45–65 years	[88]
	EA (50 µM) on oxLDL-induced proliferation	24 h	Rat thoracic smooth muscle cells	[106]
	UroB (0.1–10 μM)	24 h	Human THP-1 macrophages	[107]
Cholesterol Efflux ↑	PC (10–26 µM)	20 h	Murine J774A.1 macrophages	[108]
	EA (1–5 µM)	24 h	Murine J774A.1 macrophages	[109]
	UroB (0.1–10 µM)	24 h	Murine J774A.1 macrophages	[107]
Cholesterol Biosynthesis ↓	PC (15 or 30 µM)	20 h	Murine J774A.1 macrophages	[110]
Plasma Lipids ↓	UroA (3 mg/kg/day)	12 weeks	Adult Wister rats	[111]
Fatty Acid Oxidation ↓	UroA (250–2000 mg/day)	28 days	Human: healthy individuals (61–85 years)	[112]
Inflammatory cytokines/markers ↓	PC (40 mg/kg/day)	3 days	Sprague-Dawley rats	[113]
	EA (5–20 µM)	2 h and then 150 μg/mL oxLDL for 24 h	Human umbilical cord endothelial cells	[114]
	UroA (18 µM)	4–12 h	TNF-α-activated human aortic endothelial cells	[94]
	UroB (2.5–5 mg/kg/day)	2 weeks	Adult male Sprague Dawley rats	[115]
Autophagy/Mitophagy and mitochondrial health ↑	UroA (250–2000 mg/day)	4 weeks	Human: Healthy individuals (61–85 years)	[112]
Endothelial function ↑	PC (195 mg/day)	20 weeks	Human: Healthy individuals aged 45–65 years	[88]
	UroA (0.5–5 µM)	24 h	oxLDL-treated human aortic endothelial cells	[116]
Plaque lipid deposition ↓	UroB (10 mg/kg/day)	14 days	Male Apo-E^−/−^ mice	[107]

↑, increased/improved; ↓, decreased; ApoE^−/−^, apolipoprotein E deficient mice; EA, ellagic Acid; oxLDL, oxidized low density lipoprotein; PC, Punicalagin; TNF-α, tumour necrosis factor-α; UroA, urolithin A; UroB, urolithin B.

## Data Availability

Not applicable.

## Abbreviations

ABC: ATP-binding cassette transporter; ACAT1, acyl-CoA acyltransferase-1; Apo, apolipoprotein; ApoE^−/−^, apolipoprotein E deficient; AVCD, atherosclerotic cardiovascular disease; CCL, C-C motif ligand; CD36, cluster of differentiation 36; CE, cholesteryl ester; CETP, cholesteryl ester transfer protein; CVD, cardiovascular disease; CXC, C-X-C motif ligand; EA, ellagic acids; EC, endothelial cells; ECM, extracellular matrix; eNOS, endothelial nitric oxide synthase; ERK, extracellular signal-regulated kinase; ET, elagitannins; FFA, free fatty acids; HDL, high-density lipoprotein; HUVEC, human umbilical cord endothelial cells; ICAM-1, intercellular adhesion molecule-1; IFN, interferon; IL, interleukin; Keap1, Kelch-like ECH-associated protein 1; LDL, low-density lipoprotein; LDLr, LDL receptor; LOX-1, lectin-like oxLDL receptot-1; LPS, lipopolysaccharide; MCP-1, monocyte chemotactic protein-1; MI, myocardial infarction; NADPH, nicotinamide adenine dinucleotide phosphate; Nox4, NADPH oxidase 4; NAFLD, non-alcoholic fatty liver disease; NASH, non-alcoholic steatohepatitis; NF-κB, nuclear factor kappa-light-chain-enhancer of activated B cells; Nrf2, nuclear factor erythroid 2-related factor 2; oxLDL, oxidized LDL; PC, punicalagin; RCT, reverse cholesterol transport; ROS, reactive oxygen species; RNA-seq, RNA-sequencing; RNS, reactive nitrogen species; SMC, smooth muscle cells; SR-A1, scavenger receptor type 1; SR-B1, scavenger receptor class B type 1; TFEB, transcription factor EB; TG, triacylglycerol; TNF, tumour necrosis factor; Uro, urolithins; VCAM-1, vascular cell adhesion molecule-1; VSMC, vascular smooth muscle cells.
